# Effect of dapsone alone and in combination with intracellular antibiotics against the biofilm form of *B. burgdorferi*

**DOI:** 10.1186/s13104-020-05298-6

**Published:** 2020-09-29

**Authors:** Richard I. Horowitz, Krithika Murali, Gauri Gaur, Phyllis R. Freeman, Eva Sapi

**Affiliations:** 1 HHS Babesia and Tick-borne Pathogens Subcommittee, Washington, DC 20201 USA; 2 Hudson Valley Healing Arts Center, 4232 Albany Post Road, Hyde Park, NY 12538 USA; 3 Department of Biology and Environmental Science, University of New Haven, West Haven, CT USA

**Keywords:** Lyme disease, *Borrelia burgdorferi*, Biofilm, Dapsone

## Abstract

**Objective:**

Lyme disease is a tick-borne, multisystemic disease caused by *Borrelia burgdorferi*. Standard treatments for early Lyme disease include short courses of oral antibiotics but relapses often occur after discontinuation of treatment. Several studies have suggested that ongoing symptoms may be due to a highly antibiotic resistant form of *B. burgdorferi* called biofilms. Our recent clinical study reported the successful use of an intracellular mycobacterium persister drug used in treating leprosy, diaminodiphenyl sulfone (dapsone), in combination therapy for the treatment of Lyme disease. In this in vitro study, we evaluated the effectiveness of dapsone individually and in combination with cefuroxime and/or other antibiotics with intracellular activity including doxycycline, rifampin, and azithromycin against *Borrelia* biofilm forms utilizing crystal violet biofilm mass, and dimethyl methylene blue glycosaminoglycan assays combined with Live/Dead fluorescent microscopy analyses.

**Results:**

Dapsone, alone or in various combinations with doxycycline, rifampin and azithromycin produced a significant reduction in the mass and protective glycosaminoglycan layer and overall viability of *B. burgdorferi* biofilm forms. This in vitro study strongly suggests that dapsone combination therapy could represent a novel and effective treatment option against the biofilm form of *B. burgdorferi*.

## Introduction

Lyme disease is the number one vector-borne illness in the United States caused by *B. burgdorferi* species and transmitted via the bite of Ixodes ticks [1–3]. Successful frontline treatments for early Lyme disease involve using antibiotics including doxycycline, amoxicillin, cefuroxime axetil, and ceftriaxone [4–7]. Although standard antibiotic therapy is effective in most cases of early Lyme disease [5], CDC reports suggest that greater than 10–20% of Lyme patients who have been treated for an *Erythema migrans* (EM) rash, a classical early manifestation of Lyme disease, continue to experience symptoms of fatigue, musculoskeletal pain, and cognitive impairment despite appropriate treatment [8–13].

Several theories to explain persistent symptoms have been suggested, including immune evasion in privileged sites [14], antigenic variation [15], persistent antigenic stimulation [16], biofilm formation [17, 18] and *B. burgdorferi* persister cells, a highly resistant bacterial form which may protect the bacteria from antibacterial therapy. *B. burgdorferi* can exist in spirochetal, round body forms, intracellularly, as well as in newly discovered biofilm forms [4, 19–29]. Previous data suggested that standard and some newly discovered antibiotics for Lyme disease can be very effective in eliminating spirochetal, round body, intracellular and antibiotic tolerant persister cells [4, 20, 25–27] but have little effect on biofilm forms [24, 30]. Persisters are multi-drug tolerant cells present in significant numbers in biofilms [27, 29], and the importance of *Borrelia* biofilms has been highlighted in autopsy tissues from a well-documented Lyme disease patient [31]. Therefore, to successfully treat Lyme disease, there is an urgent need to find an agent or combination of antimicrobial agents which can efficiently eliminate resistant biofilm forms of *B. burgdorferi.*

Dapsone combination therapy (DDS CT) is clinically effective as a novel drug regimen for the treatment of chronic Lyme disease as reported in 100 patients who had previously failed commonly prescribed antibiotic therapies [32]. A recent retrospective study among a larger group of 200 patients also found that dapsone (DDS) combined with other intracellular antibiotics including doxycycline, rifampin, and/or azithromycin was effective in reducing 8 major Lyme disease symptoms [33, 34]. Although dapsone combination therapy was clinically effective, its efficacy against different morphological forms of *B. burgdorferi* in culture had not yet been fully studied. In a previous 2017 study, Feng et al. compared the efficacy of sulfa drugs including dapsone, sulfamethoxazole, sulfachlorpyridazine, and assessed their combinations along with commonly prescribed Lyme antibiotics for their activity against *B. burgdorferi* “persisters” and found that dapsone was the most active drug among the 3 sulfa drugs [35]. However, this study did not evaluate the effect of any of the sulfa drugs on attached *B. burgdorferi* biofilm. We therefore designed an in vitro study using dapsone as a single drug and/or in combination with cefuroxime axetil and/or other antibiotics with intracellular activity including doxycycline, rifampin and azithromycin in order to find the most effective combinations to effectively eliminate resistant biofilm forms of *Borrelia burgdorferi*.

## Main text

### Methods

#### Bacteria culture conditions

Low passage isolates of *B. burgdorferi,* B31 strain were obtained from ATCC (#35,210, Manassas, VA) and cultured in Barbour-Stoner-Kelly H (BSK-H) media (Sigma, St Louis, MO) supplemented with 6% rabbit serum (Pel-Freez®, Rogers, AR). The stock cultures were maintained in sterile 15 ml glass tubes and incubated at 33 °C with 5% CO_2_ in the absence of antibiotics. Spirochetes for surface attached biofilms were seeded at 5 × 10^6^ cells/ml in 4-well Permanox chamber slides (Thermo Scientific, Waltham, MA) or in 48-well sterile tissue culture plates (BD Falcon, Franklin Lakes, NJ) for 5 days to establish attached biofilm form. Floating spirochetal cells and aggregates from the supernatant were removed to ensure only surface attached biofilms would be analyzed.

#### Antibiotics

All antibiotics were prepared in standard 1 × phosphate buffered saline solution (PBS) and sterilized using 0.2 µm filter unit (EMD Millipore, Billerica, MA). As a negative control, 1 × PBS was used which was the diluent for antimicrobial compounds studied.

*Crystal violet biofilm,* Baclight *LIVE/DEAD viability, Dimethylmethylene blue glycosaminoglycan (*DMMB) assays.

The effect of the antimicrobial agents on *B. burgdorferi* biofilm mass and viability was evaluated by using a crystal violet assay and *LIVE/DEAD* microscopic analyses respectively as described earlier [30]. The effectiveness of the antibiotic combinations on the *B. burgdorferi* biofilms was also determined by quantifying the biofilm polysaccharide matrix content, glycosaminoglycans (GAG) as described [36].

#### Statistical analysis

Quantitative results were analyzed using the median value of all the readings from antimicrobial screens in addition to a two-tailed Student’s t -test (Microsoft Excel, Redmond, WA, USA). All experiments were performed a minimum of five independent times with at least four replicates per each experimental condition (N = 20). Each experiment was repeated by two different scientists (GG and KM) and statistical analyses were conducted independently by a third (ES).

## Results

In this study, we compared and evaluated the antimicrobial effect of dapsone along with the other clinically tested antibiotics (doxycycline, rifampin, azithromycin, cefuroxime) on the growth and viability of attached *B. burgdorferi* biofilms using standard crystal violet biofilm mass and dimethylmethylene blue glycosaminoglycan assays combined with BacLight Live/Dead microscopic analysis. As in the previous studies from our group and others [30, 35], we tested two different antibiotic concentrations (10 µM and 50 µM) against attached *B. burgdorferi* biofilm structures. The 10 µM in vitro concentration corresponds well to the achievable serum level after administration of the antibiotics tested in this study [37–41]. Recent studies showed however that higher 50 µM concentrations for these antibiotics could be very effective against persister cells [27, 35], therefore this higher concentration was also tested.

First, the effectiveness of the single and combination antibiotic treatments on attached *Borrelia* biofilm was quantified by crystal violet biofilm mass assay after 72 h treatments with various antibiotics. The most significant results were achieved with individual and combination treatments with dapsone, as listed in Table 1. The best reduction in biofilm mass by a single antibiotic was achieved with dapsone at 10 µM and 50 µM concentrations resulting in 69% and 58% residual viability respectively, when compared to the PBS treated control (p value < 0.01). Rifampin at both 10 µM and 50 µM concentrations also resulted in a significant decrease in biofilm mass (76% and 60% respectively, p values < 0.01) compared to the PBS treated negative control samples, although not quite as effective as dapsone alone at comparable concentrations. Treatments with other single antibiotics including doxycycline, cefuroxime, and azithromycin were less effective and, in some cases, even increased biofilm size when compared to the PBS treated control (Table 1).

**Table 1:** Effect of different antibiotic treatments on attached *B. burgdorferi* biofilm mass at 10 µM and 50 µM concentration evaluated with crystal violet biofilm assay after 72 h

Antibiotics single	10 µM residual % ± %SD	50 µM residual % ± %SD	Antibiotics dual combinations	10 µM residual % ± %SD	50 µM residual ± %SD
Control (PBS)	100%	100%	Control (PBS)	100%	100%
DDS	69% ± 5.1	58% ± 4.7	DDS + DOXY	68% ± 5.3	65% ± 4.6
DOXY	107% ± 8.2	103% ± 11.6	DDS + RIF	82% ± 7.6	71% ± 5.6
RIF	76% ± 4.3	60% ± 8.9	DDS + AZ	106% ± 9.1	93% ± 7.3
CEF	109% ± 8.6	102% ± 9.5	DDS + CEF	83% ± 7.3	101% ± 10.3
AZ	101% ± 7.4	91% ± 7.9	DOXY + RIF	73% ± 5.8	74% ± 6.5

Antibiotics triple combinations	10 µM residual % ± %SD	50 µM residual % ± %SD	Antibiotics quadruple combinations	10 µM residual % ± %SD	50 µM residual % ± %SD
Control (PBS)	100%	100%	Control (PBS)	100%	100%
DDS + DOXY + RIF	78% ± 5.8	52% ± 4.1	DDS + DOXY + RIF + CEF	72% ± 5.2	79% ± 6.2
DDS + DOXY + CEF	65% ± 4.2	60% ± 8.8	DDS + DOXY + RIF + AZ	78% ± 5.4	58% ± 3.5
DDS + DOXY + AZ	80% ± 7.7	72% ± 6.5	DDS + RIF + AZ + CEF	81% ± 6.8	68% ± 5.5
DDS + RIF + AZ	69% ± 4.2	77% ± 8.5	DOX + RIF + AZ + CEF	98% ± 8.5	82% ± 5.1
DDS + RIF + CEF	70% ± 5.6	62% ± 5.6			

Table summarizes the mean % of residual viability with ± SD compared to the PBS treated control. N = 20

*DDS* dapsone, *DOXY* doxycycline, *RIF* rifampin, *CEF* cefuroxime, *AZ* azithromycin

The most effective dual combination was dapsone + doxycycline at both 10 µM and 50 µM concentrations (68% and 65% residual viability respectively) significantly decreasing biofilm size compared to the PBS treated control (p value < 0.01; Table 1). However, when compared to dapsone alone treated samples, this dual combination was not more effective than dapsone alone (*p* value > 0.05).

The triple combination treatment of dapsone + doxycycline + rifampin (52% residual viability) and quadruple combination of dapsone + doxycycline + rifampin + azithromycin (58% residual viability) treatments both at 50 µM concentration were the most effective when compared to the PBS treated control (*p* values < 0.01), however, the effect of three and four drug combinations at 50 µM was not significantly better than the 50 µM concentration treatments of dapsone alone or dapsone + doxycycline (*p* values > 0.05).

**Fig. 1:**
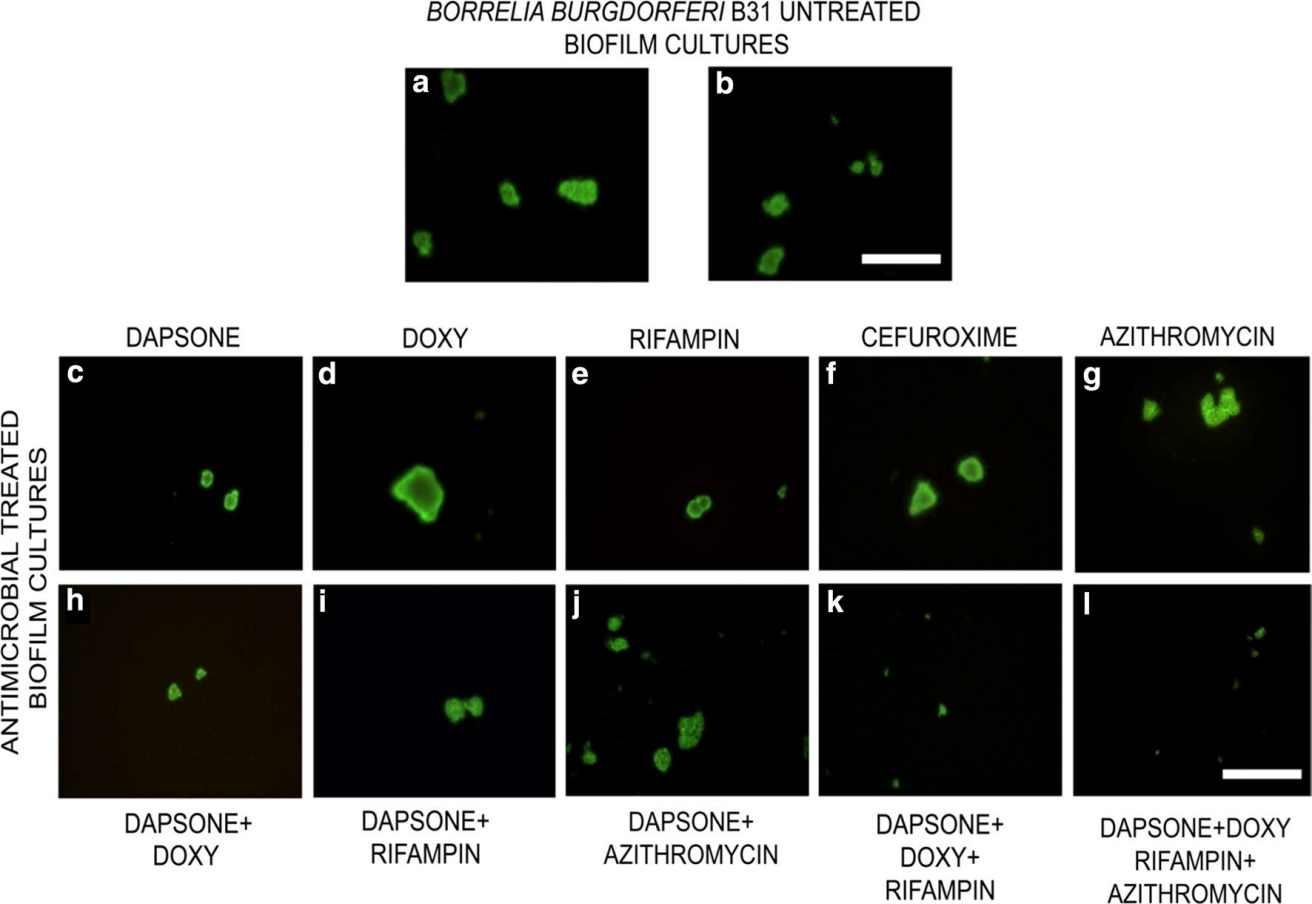
Representative Live/Dead images of the attached *B. burgdorferi* biofilms following a 72 h treatment with different antimicrobial agents at 10 µM. Biofilms were analyzed by *LIVE/DEAD* assay as outlined in the Methods and representative images were taken at 100X magnification. Scale bar: 100 μm

Crystal violet biofilm assay only measures the cellular mass (both live or dead) and does not provide information about the viability and the individual sizes of the antibiotic treated biofilm aggregates. Therefore, Live/Dead fluorescent microscopy techniques were used to visualize the effect of the most effective single and combination treatments after 72 h with different antibiotics and represented images are presented in Fig. 1. Biofilm cultures treated only with PBS show live (green) and compact morphology (Fig. 1a, b). For single and dual antibiotic treatments, the microscopy data were in good agreement with the crystal violet data. For example, it confirmed that single treatment dapsone (Fig. 1c) and dual treatment dapsone + doxycycline (Fig. 1h) were indeed effective in reducing *Borrelia* biofilm size, resulting in very small aggregates (less than 20 µm aggregates [Fig. 1c]).

However, for triple and quadruple antibiotic treatments, such as dapsone + doxycycline + rifampin and dapsone + doxycycline + rifampin + azithromycin, the microscopy images suggest a more significant effect of these triple and quadruple combinations than dapsone or dapsone + doxycycline as demonstrated by very small (less than 10 µm) and disorganized biofilms structures (Fig. 1k, l).

In order to further evaluate the effectiveness of the antibiotics on the attached biofilm form of *B. burgdorferi*, the amount of the protective layers of biofilm polysaccharide matrix of these aggregates were measured using the DMMB glycosaminoglycan (GAG) assay before and after 72 h antibiotic treatments. Biofilms treated with negative control, PBS, showed no reduction in the amounts of GAG, when compared with the untreated biofilm control. The most significant results with the different single and combination of antibiotics are summarized in Table 2. Single antibiotic treatments at 10 µM of rifampin and doxycycline were the most effective resulting in 75% and 76% residual GAG amounts compared to the PBS treated control (*p* value < 0.01). However, at 50 µM, dapsone showed the most significant effects with 68% residual GAG amounts of *Borrelia* biofilm compared to the untreated control (*p* value < 0.01). For combination therapies, dapsone + doxycycline + rifampin at 10 µM and dapsone + doxycycline + rifampin + azithromycin at 50 µM were the most effective agents when compared with the untreated control with 69% and 62% residual GAG amounts respectively. The 50 µM quadruple combination data were found to be significant not just in comparison to the negative control but to any single treatment result (*p* value < 0.05).

**Table 2:** Effect of different antibiotic treatments on attached *B. burgdorferi* biofilm glycosaminoglycan (GAG) content at 50 µM concentration evaluated with Dimethylmethylene Blue Assay for Glycosaminoglycan after 72 h

Antibiotics	10 µM GAG % ± %SD	50 µM GAG % ± %SD
Control (PBS)	100%	100%
DDS	79% ± 5.8	68% ± 3.6
DOXY	76% ± 4.2	73% ± 7.2
RIF	75% ± 6.3	70% ± 5.8
CEF	78% ± 5.2	76% ± 7.4
AZ	82% ± 6.6	79% ± 8.2
DDS + DOXY	70% ± 8.2	69% ± 5.3
DDS + DOXY + RIF	69% ± 4.2	70% ± 7.3
DDS + DOXY + RIF + AZ	75% ± 6.8	62% ± 5.4

The table summarizes that mean % of the residual GAG amounts with ± SD compared to the PBS treated control. N = 20

*DDS* dapsone, *DOXY* doxycycline, *RIF* rifampin, *CEF* cefuroxime, *AZ* azithromycin

## Discussion

The major findings from this study are that dapsone, as a single drug and in combination with doxycycline and doxycycline + rifampin as well as doxycycline + rifampin + azithromycin had the most significant effect in reducing the mass and viability as well the protective mucopolysaccharide layers of *B. burgdorferi* biofilm. These findings might explain at least in part its clinical efficacy seen in recent DDS CT trials [32–34].

In order to eliminate the most resistant forms of *B. burgdorferi,* there is a need for safe and effective drugs that are able to eliminate all morphological forms of *B. burgdorferi* including attached biofilm forms [24, 30]. Our recent clinical studies reported that DDS combination therapy [DDS with rifampin and a tetracycline (doxycycline, minocycline) and/or a macrolide (azithromycin, clarithromycin) and/or cephalosporin (cefuroxime axetil)] improved symptoms of fatigue, muscle/joint pain, neuropathy, disturbed sleep, cognitive complaints, and sweats and/or flushing [32–34].

A recent study [35] evaluated DDS for activity against persisters and found it was not only the most active sulfa drug but exhibited in vitro superiority to other antibiotics including rifampin, azithromycin, and minocycline. Unfortunately, these researchers did not evaluate DDS combinations against attached *B. burgdorferi* biofilm, the most antibiotic resistant form in vitro [24, 30] and a dominant form in a human autopsy study from a Lyme disease patient [31]. Furthermore, a recent mouse model study [42] showed that the biofilm like microcolony and stationary phase planktonic forms (free cells) caused more severe Lyme arthritis with an earlier onset of inflammation and joint swelling than the log phase spirochetes. Therefore, addressing biofilm forms is vital, and for that reason in this study, we added several assays to evaluate dapsone alone and in combination on *B. burgdorferi* biofilms. We found that dapsone as a single agent is very effective in reducing the attached biofilm forms, and that dapsone combination therapy (DDS CT) with a tetracycline, rifampin and/or macrolide had a more significant effect on reducing the mass and viability as well the protective mucopolysaccharide layers of highly resistant *B. burgdorferi* biofilm.

## Conclusion

Results from this study verify that dapsone alone and in combination with other antibiotics is effective in reducing the antibiotic resistant biofilm forms of *B. burgdorferi* and support the in vitro effectiveness of DDS CT. Two prior published retrospective studies using DDS CT also showed clinical efficacy in relieving eight major Lyme symptoms. Considering the worldwide spread of borreliosis and significant numbers of individuals with persistent Lyme symptoms, prospective randomized trials are urgently required to evaluate the clinical efficacy of DDS CT.

## Limitations

This study evaluated the antibiotic sensitivity of the B. burgdorferi B31 laboratory strain and needs to be repeated with other Bb sensu lato strains as well to evaluate efficacy.

## Data Availability

The datasets used and/or analyzed during the current study are available from Dr. Eva Sapi, University of New Haven, on reasonable request.

## Abbreviations

CDC: Centers for Disease Control and Prevention
DDS: Dapsone
DDS CT: Dapsone combination therapy
DMMB: Dimethylmethylene blue glycosaminoglycan
EM: Erythema migrans
GAG: Glycosaminoglycan
PBS: Phosphate buffered saline
